# From Imagination to Immersion: The Impact of Augmented Reality Instruction on Musical Emotion Processing: An fNIRS Hyperscanning Study

**DOI:** 10.3390/brainsci16010066

**Published:** 2025-12-31

**Authors:** Qiong Ge, Jie Lin, Huiling Zhou, Jing Qi, Yifan Sun, Jiamei Lu

**Affiliations:** 1 School of Psychology, Shanghai Normal University, Shanghai 200234, China; 1000497860@smail.shnu.edu.cn (Q.G.); 1000550026@smail.shnu.edu.cn (H.Z.); 1000497863@smail.shnu.edu.cn (Y.S.); 2 School of Music, Zhejiang Normal University, Jinhua 321004, China; livia888899@zjnu.cn; 3 Faculty of Social Sciences and Liberal Arts, UCSI University, Kuala Lumpur 56000, Malaysia; 1002265679@ucsiuniversity.edu.my

**Keywords:** augmented reality, visual mental imagery, music emotion processing, fNIRS hyperscanning, Chinese pipa music

## Abstract

**Background**: This study addresses a common challenge in music education: students’ limited emotional engagement during music listening. **Objectives**: This study compared two teaching methods—externally guided augmented reality (AR) integration and internally generated simulation—in terms of their neural and behavioral differences in guiding students’ visual mental imagery and influencing their musical affect processing. **Methods**: Using Chinese Pipa music appreciation as our experimental paradigm, we employed fNIRS hyperscanning to record inter-brain synchronization (IBS) during teacher–student interactions across three instructional conditions (AR group, *n* = 27; visual imagery group, *n* = 27; no-instruction group, *n* = 27), while simultaneously assessing students’ performance in music–emotion processing tasks (emotion recognition and experience). **Results**: At the behavioral level, both instructional methods significantly enhanced students’ ability to differentiate emotional valence in music compared to the control condition. Crucially, the AR approach demonstrated a unique advantage in augmenting emotional arousal. Neurally, both teaching methods significantly enhanced IBS in brain regions associated with emotion evaluation (lOFC) and imaginative reasoning (bilateral dlPFC). Beyond these shared neural correlates, AR instruction specifically engaged additional brain networks supporting social cognition (lFPC) and multisensory integration (rANG). Furthermore, we identified a significant positive correlation between lFPC-IBS and improved emotional arousal exclusively in the AR group. **Conclusions**: The visual imagery group primarily enhances emotional music processing through neural alignment in core emotional brain regions, while augmented reality instruction creates unique advantages by additionally activating brain networks associated with social cognition and cross-modal integration. This research provides neuroscientific evidence for the dissociable mechanisms through which different teaching approaches enhance music–emotion learning, offering important implications for developing evidence-based educational technologies.

## 1. Introduction

Music education aims to cultivate students’ emotional processing abilities and aesthetic literacy [1]. However, the widespread phenomenon of “lack of emotional” in teaching practice [2]—where students possess normal auditory perception yet struggle to establish effective connections between auditory input and internal emotional responses—severely limits music’s deeper educational functions in emotional regulation and empathy development [3,4]. Therefore, developing effective teaching strategies that simultaneously enhance emotional recognition accuracy and deepen emotional experiences has become an urgent need in contemporary music education.

Traditional music education has yet to establish systematic teaching methods for emotional processing, with strategy selection exhibiting strong subjective arbitrariness. This has become a bottleneck constraining students’ development of musical emotional processing abilities [5]. To effectively address this issue, academic research has gradually shifted its focus toward the intrinsic cognitive processes and neural mechanisms underlying music-induced emotions, seeking to explore potential pathways for teaching optimization at their root [6]. Among various emotion-inducing mechanisms, visual mental imagery is regarded as a core component—the capacity of music to actively evoke images and scenes in the listener’s mind [6]. This phenomenon essentially represents the brain’s active simulation of perceptual experiences [7]. However, how to effectively translate this theoretical mechanism into actionable teaching pathways remains an unaddressed theoretical gap in current research.

To this end, this study distinguishes two generative pathways based on the mechanism of visual mental imagery: internally generated simulation and externally guided integration. Internally generated simulation primarily relies on the activation of an individual’s internal resources (such as autobiographical memory) [8]. It first uses external linguistic cues to induce students to form internal visual images matching the emotional content of the music. Subsequently, it employs this active mental simulation to experience the music, thereby enhancing their emotional engagement. However, its effectiveness is significantly influenced by an individual’s visual imagery ability, memory store capacity, and emotional schema strength [9,10]. Consequently, for individuals with low visual imagery ability or weak autobiographical memory stores, both the efficiency of the internal simulation process and the depth of emotional experience may be substantially limited.

The externally guided integration pathway is grounded in the theory of “extended cognition,” which posits that cognition can leverage the external environment as scaffolding [11]. This study incorporates augmented reality technology to construct visual scenes matching musical emotions, providing learners with embodied external emotional anchors [12]. The efficacy of this technology has received preliminary support from neuroscience evidence [13,14]. Compared to internally generated pathways, externally integrated pathways guided by augmented reality (AR) hold promise for significantly reducing intrinsic representational variability caused by individual ability differences [15] by providing unified structured visual input. By transforming abstract emotions into shared embodied experiences, this pathway may demonstrate more reliable pedagogical advantages.

To objectively evaluate the practical effectiveness of two teaching methods, this study employs a neuroeducation research paradigm. By measuring brain activity in teachers and students within real classroom settings, it systematically explores the underlying neural mechanisms of different instructional approaches. The research design is grounded in contemporary teaching theory: teachers, students, and teaching materials serve as the three core emotional anchors in the teaching process. The strength of their emotional connection directly influences the implementation outcomes of affective teaching [16,17]. Functional near-infrared spectroscopy (fNIRS) hyperscanning technology provides a feasible research pathway to achieve this objective. This technique not only enables simultaneous recording of dynamic brain activity during teacher–student interactions but is also particularly suited for capturing brain responses in natural teaching settings due to its robust tolerance to motion artifacts [18] and effective monitoring of auditory and higher-order cognitive-related cortical regions [19]. Its core observational metric—interbrain synchrony (IBS)—has been demonstrated as a potential neural marker for successful communication and learning [20,21]. For instance, neural synchronization between teachers and students in specific brain regions can predict student learning outcomes [22]. A series of studies have significantly advanced our understanding of the neural basis of teaching processes through this approach [23,24]. Consequently, fNIRS hyperscanning serves as an ideal tool for investigating the neural interaction mechanisms between teachers and students under different teaching methods.

Neuroimaging studies consistently indicate that the prefrontal cortex (PFC) and its associated brain regions play a critical role in emotional processing. Specifically, the frontopolar cortex (FPC; BA10) participates in the cognitive regulation of emotions, responsible for coordinating the allocation of attention to external stimuli and maintaining internally generated cognitive processes [25,26]; the dorsolateral prefrontal cortex (dlPFC; BA9/46) is essential for higher-order executive functions such as logical reasoning and imagination in emotional contexts [27]; The orbitofrontal cortex (OFC; BA11) is closely associated with the processing of interoceptive emotional signals and subjective emotional experiences [28]. Additionally, the right temporoparietal junction (TPJ, BA39, BA40) serves as a core hub of the social cognitive network, playing a crucial role in multimodal information integration, intention comprehension, and social interaction [22]. Especially, the right angular gyrus plays a crucial role in cross-modal information integration, episodic memory retrieval, and self-referential processing [29]. Given the critical functions of these brain regions in musical emotion processing and teaching interactions, this study selected subregions of the PFC and the right TPJ as regions of interest to systematically examine neural activity differences elicited by different teaching methods.

The activity in these brain regions provides a foundation for understanding the neural mechanisms underlying instructional interventions: activation in the dlPFC may reflect involvement in visual mental imagery construction and logical reasoning, while activation in the OFC may be associated with interoceptive experiences during musical emotion processing. Together, these form the potential neural basis for successfully inducing visual mental imagery and influencing emotional processing. Compared to internally generated imagery pathways reliant on personalized internal imagination, AR instruction provides an objective, unified “anchor” for teacher and student attention and cognition through its immersive, standardized shared visual space. This significantly reduces interpretation ambiguity and cognitive load. Therefore, AR instruction may not only activate the aforementioned brain regions associated with the dlPFC and OFC but also potentially offer unique advantages in key brain areas crucial for enhancing cross-modal information integration (rANG) or sustaining attention to external stimuli and internally generated cognitive processes (FPC).

Based on the current state of research, the following three key questions remain to be clarified: (1) At the behavioral level, whether there are significant differences between AR-guided external integration pathways and teacher-language-guided internal generation pathways in enhancing the efficiency of musical emotion processing; (2) At the neural level, do these two instructional conditions induce fundamentally different patterns of brain activity—manifesting as differences in activation intensity within the same brain network, or relying on distinct functional networks? (3) If behavioral advantages exist, do they possess specific neural underpinnings? That is, does superior emotional learning correspond to more synergistic and efficient brain network integration patterns?

To address the aforementioned issues, this study recruited college student participants to conduct an emotion teaching experiment based on traditional Chinese pipa music (see Supplementary Material S1 for a detailed introduction to pipa music). We selected purely instrumental material performed on a single instrument (the pipa) to eliminate interference from additional variables such as multi-instrument timbres and lyrics. This music constructs abstract artistic conception through unique timbres, performance techniques (such as plucking and tremolo), and stark dynamic contrasts. It tends to evoke a phenomenon of “lack of emotional “in listeners with limited musical appreciation experience. Consequently, it serves as an ideal musical paradigm for testing whether two teaching interventions can effectively guide visual musical imagery and emotional processing. During participant screening, we strictly controlled their level of preference for pipa music and frequency of daily exposure, ensuring both were at a moderately low level. During the course implementation, we systematically compared three teaching conditions: the Visual Mental Imagery Group (internally generated simulation teaching), the AR Group (externally guided integration teaching), and a control group without instruction. Concurrently, we recorded brain activity in the prefrontal cortex and right temporo-parietal junction of both teachers and students using functional near-infrared spectroscopy hyperscanning (fNIRS hyperscanning) to systematically compare the behavioral benefits and neural foundations of different teaching methods. To ensure the validity of the experimental comparison, this study strictly controlled the consistency of visual imagery content conveyed between the AR group and the visual mental imagery group. The key difference between the two groups lies in the distinct methods of visual mental imagery guidance (external guidance integration vs. internally generated simulation), rather than the stimulus content itself. Additionally, both groups employed “delayed cross-modal integration,” first guiding the generation of visual mental imagery before listening to music. The aim was to compare the efficiency of these two imagery generation pathways in the core task of “generating and binding to music,” focusing on their potential as transferable teaching strategies rather than testing real-time multisensory enhancement effects. Based on the aforementioned theoretical framework, we propose the following hypotheses:

**H1.**  *Regarding behavioral performance in musical emotion processing, we predict both teaching groups will significantly outperform the control group, with AR-guided external integration pathways potentially demonstrating optimal effects.*

**H2.**  *In teaching interactions, we predict both instructional conditions will induce stronger inter-brain synchronization (IBS) between teachers and students, particularly in regions associated with emotional integration and social interaction (e.g., prefrontal cortex and temporo-parietal junction), with the AR group exhibiting the highest IBS levels.*

**H3.**  *We predict that the degree of enhanced inter-brain synchronization (IBS) between teachers and students will positively predict the improvement in their music–emotion processing performance, with this relationship being most pronounced in the AR-guided external integration pathway.*

## 2. Method

### 2.1. Participants and Design

Using G*Power 3.1.9.2 software to estimate the sample size required for this study [30], we set the effect size to 0.25, the significance level α = 0.05, and the power to 0.8 [31]. With a 2 (musical affect valence: positive vs. negative) × 3 (teaching methods: no instruction vs. visual mental imagery teaching vs. AR teaching) mixed design. At least 66 participants were required, consistent with previous studies on teaching interactions. Assigning 20–24 teacher–student pairs per condition would achieve an effect size of 0.20–0.25 [22,24]. This study recruited 83 Chinese university students, of whom two were excluded due to missing marks caused by poor device connectivity. Consequently, 81 participants (52 females; age: 22.58 ± 2.47) completed the experiment. Teachers and learners were unfamiliar with each other prior to the study. Learners were randomly assigned to one of three groups: (1) Control group (no instruction): 27 learners, 17 females, age: 22.15 ± 2.55; (2) Visual imagery instruction group: 27 learners, 17 females, age: 23.15 ± 2.7; AR Instruction Group: 27 learners, 18 females, age: 22.44 ± 2.1). The experiment recruited three music teachers (age: 27.33 ± 1.15, all female) with over 5 years of teaching experience. All teachers underwent standardized training prior to the experiment (including a uniform teaching process, standardized teaching content, and neutral tone of voice). All three teachers participated in experiments under all three teaching conditions. Under each condition, each teacher was randomly assigned to be paired with nine students. This resulted in a total of 81 dyads. All participants had normal hearing, normal vision or corrected vision, no psychiatric disorders, were non-music majors, and had not received professional music training (for more than one year) outside of school music classes. Preference for pipa music and daily exposure to pipa music were controlled at or below the moderate level (≤4) on a 7-point scale. All participants (teachers and students) signed informed consent forms prior to the experiment. Following the experiment, each student participant received compensation of 40 RMB, while the three participating teachers received fixed remuneration commensurate with their teaching responsibilities. This study was approved by the Ethics Committee of Shanghai Normal University.

### 2.2. Materials

Materials include a demographic questionnaire, visual mental imagery ratings, a music emotion processing scale, musical materials and assessments, AR video production and presentation. All materials are in Mandarin.

#### 2.2.1. Demographic Questionnaire

The demographic questionnaire collects participants’ gender, grade level, age, major, as well as their preferences for pipa music and frequency of daily exposure to pipa music.

#### 2.2.2. Musical Emotional Processing Scale, MEP

The assessment of music emotion recognition and music emotion experience was measured using a self-report scale based on the two-dimensional model of valence and arousal [32]. These two dimensions were guided and distinguished through different instructions and questioning methods [33]. Previous research indicates that sequence differences do not yield significant outcome variations [34]. Therefore, the following four questions are presented in a fixed order.

Music emotion recognition measures valence and arousal using two questions: 1. What emotional valence does this piece of music aim to convey? 2. How intense is this emotion? Select from a 7-point scale ranging from low to high (1 to 7), where 1 represents negative/calm and 7 represents positive/excited. The musical affective experience encompasses valence and arousal, measured through two questions: 1. What emotional valence did this piece of music genuinely evoke in you? 2. How intense is this emotion for you? Select from 7 levels ranging from low to high (1–7), where 1 represents negative/calm and 7 represents positive/excited. Cronbach’s α for this scale was 0.77 for the pre-teaching administration and 0.85 for the post-teaching administration.

#### 2.2.3. Visual Mental Imagery Ratings, VMI

The Visual Mental Imagery (VMI) Rating Scale comprises two questions. 1. How prevalent are visual mental images while listening to music? 2. How vivid are these visual mental images? (1 indicates almost none/fuzzy and scattered; 9 indicates very numerous/vivid and three-dimensional) [35]. Cronbach’s α for this scale was 0.76 for the pre-teaching administration and 0.9 for the post-teaching administration.

#### 2.2.4. Musical Materials and Assessment

The study utilized ten solo pipa recordings sourced from leading Chinese digital music services, including Kugou, QQ Music, and NetEase Cloud Music. Each piece was performed by recognized professional musicians and composed within the traditional pentatonic framework. A key consideration during the selection process was to maintain the authentic emotional expression of the original compositions. To approximate the realistic listening context of a music appreciation lesson, in which full works are typically presented, 120-s excerpts were created from each recording. All excerpts were normalized to a loudness range of 65–75 dB and preserved in 16-bit/44.1 kHz audio quality to ensure consistency and preserve the artistic integrity of the source material. Following established procedures for evaluating musical emotion induction [36], the selected excerpts were assessed based on two emotional dimensions—valence and arousal—to confirm their effectiveness in evoking the intended positive or negative emotional responses.

**Figure 1:**
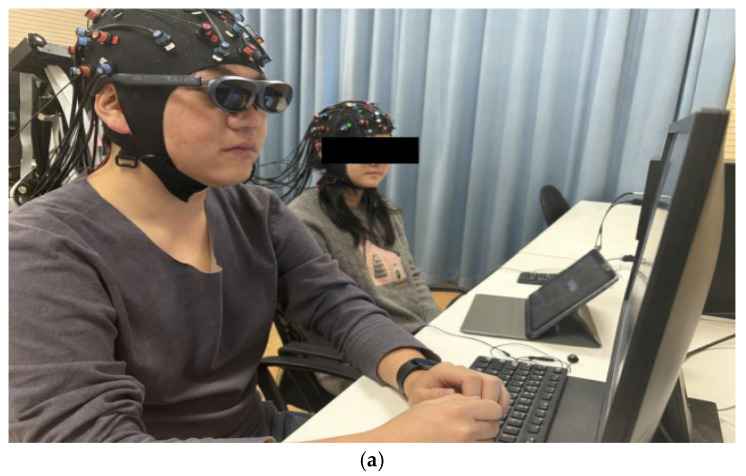

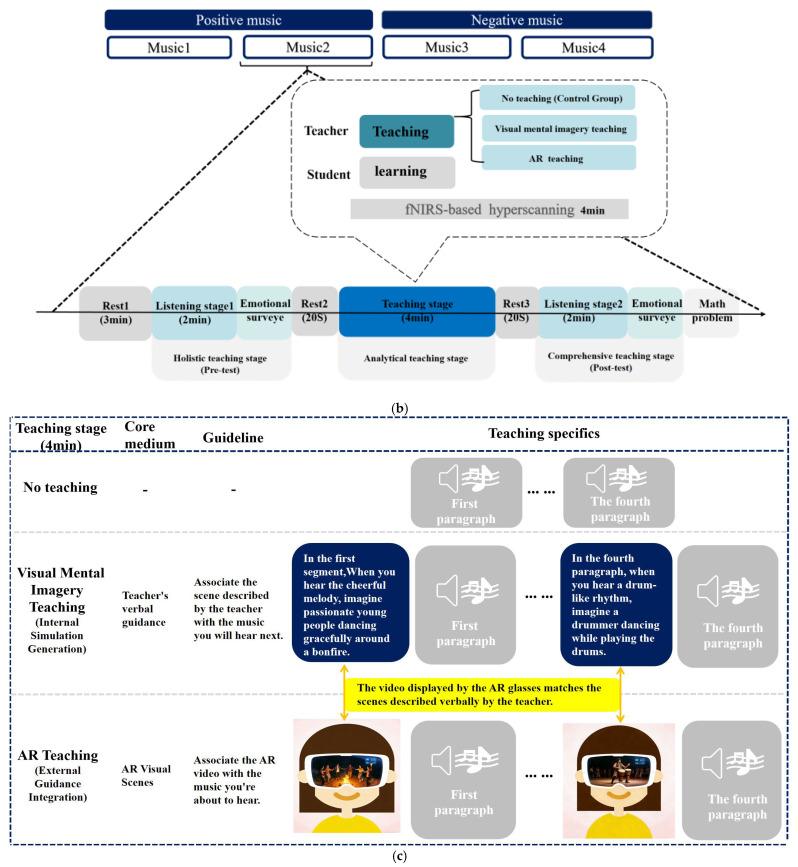
(**a**) Schematic diagram of the experimental setting. (**b**) Experimental procedure for the music appreciation class. (**c**) Specific design example for the teaching phase.

Thirty-two non-music major undergraduate students were recruited to evaluate the selected music excerpts along two emotional dimensions: emotional valence (the extent to which the music evoked positive or negative feelings) and arousal (the level of emotional intensity, ranging from calm/relaxed to excited/agitated). Ratings were collected on a 7-point Likert scale (1 = negative/calm, 7 = positive/excited). Additionally, participants rated their familiarity with each piece on a 7-point scale (1 = not familiar at all, 7 = highly familiar). Pieces rated as moderately or highly familiar were excluded from the final selection. Based on these evaluations, four pieces were ultimately selected—two positively valenced and two negatively valenced (see Supplementary Material S1.2). For the instructional phase of the study (Figure 1c), each 120-s piece was divided into four 30-s segments to facilitate teaching and analysis. The mean valence ratings were 5.47 ± 1.07 for positive music and 3.59 ± 1.33 for negative music. A significant difference in valence was observed between the two types of music, *t*(31) = 5.98, *p* < 0.001, Cohen’s d = 1.56. Both positive and negative pieces were rated above the midpoint on arousal and below the midpoint on familiarity. No statistically significant differences were found between the two music types in terms of arousal, *t*(31) = −0.70, *p* = 0.49, or familiarity, *t*(31) = −0.57, *p* = 0.58.

#### 2.2.5. AR Video Production and Presentation

In this study, AR (augmented reality) is technically implemented by projecting pre-recorded video content onto a head-mounted display device AR glasses (Rokid Max RA201, resolution 3840 × 1080 p, Hangzhou Lingban Technology Co., Ltd., Hangzhou, China), providing learners with a closed, immersive, visually guided environment. Its core design principle is to offer a highly focused external visual scaffold distinct from traditional flat screens, serving the pedagogical experimental purpose of “extended cognition”.

To develop experimental materials for the AR teaching group, we followed standardized procedures to prepare visual stimuli: (1) We selected traditional Chinese pipa music (using “The Overlord Removes His Armor” as an example; See Supplementary Material S1.2) to conduct structural and narrative analysis, dividing it into four distinct segments with pronounced emotional and plot differences: “On the Eve of War,” “Army Deployment,” “Fierce Battle,” and “Surrounded by Enemy Forces.” (2) For each musical segment, collaborated with the teaching team to deliberate and determine the core visual themes and emotional tone that align with it. For instance, the video for the “On the Eve of War” segment aims to convey an atmosphere of tension, oppression, and impending storm. Based on the established themes, searches were conducted on major video platforms (e.g., Douyin, Bilibili, Youku) using precise keyword combinations (e.g., “ancient army assembly,” “pre-battle preparations,” “dark clouds over the city”) to identify potential video clips. Selection criteria prioritized high visual alignment with the musical segment’s emotional tone, image quality, and authenticity of content. (3) All selected videos underwent standardized processing using professional editing software: A total of 4 × 4 =16 videos were ultimately chosen, uniformly cropped to 25 s, stripped of their original audio tracks, and resized to 1920 × 1080 pixels. The final videos were exported in MP4 format (H.264) and pre-stored on the iPad (iPad 9 (2021), 10.2-inch, Apple Inc., Cupertino, CA, USA) Air devices designated for the experiment.

To achieve the visual stimulus presentation of the AR teaching group on AR glasses, this study completed the transmission and display of visual signals through the following device connection scheme and parameter settings: Connecting the iPad and Rokid Station (Max+station, Hangzhou Lingban Technology Co., Ltd., Hangzhou, China) to the same Wi-Fi network, activating the iPad’s screen mirroring function to project the video signal to the Rokid Station, which then transmits it to the AR glasses. During the experiment, participants did not wear light-blocking goggles to maintain the AR glasses’ see-through functionality. While the video played, the front computer screen displayed a pure black background to prevent ambient light scattering from interfering with the AR video presentation. After video playback ended, the AR glasses switched to a pure white image to ensure the see-through function remained active, allowing participants to observe the computer screen and complete subsequent reaction-key tasks. All video transitions were controlled by the instructor on an iPad according to the lesson progression.

### 2.3. Procedure

The experiment was conducted in a quiet, acoustically isolated laboratory under consistent lighting conditions. The experimental procedure was developed using E-Prime 3.0 and executed on a 19-inch Dell computer, which automatically recorded all responses. Audio stimuli were delivered through two synchronized headphones (SONY, Tokyo, Japan, MDR-EX650AP) connected via an external audio splitter. Student participants were randomly paired with an instructor and assigned to one of the experimental groups. The experimental setup is illustrated in Figure 1a.

During the preparation phase, both instructors and student participants sat at computers, adjusted their posture, and were assisted by the experimenter in wearing the wearable fNIRS headgear (NIRx Medizintechnik GmbH, Berlin, Germany) and headphones (SONY, Tokyo, Japan, MDR-EX650AP), with volume levels calibrated. Light sources and detectors were positioned according to the headgear’s channel layout template, and light plates were adjusted until optimal signal quality was achieved. After confirming stable probe contact and acceptable signal quality with the fNIRS headcap, the AR group then donned AR glasses, securing the temples securely to the outer side of the fNIRS headcap straps. Following device setup, a secondary signal quality check is conducted. All participants proceed to the experimental phase only after confirming no additional discomfort.

The formal experiment was divided into three phases according to the standard music appreciation class teaching process (see Figure 1b).

During the holistic listening phase—the initial full-length music listening session—participants focused their gaze on a designated point on the screen throughout the music playback. Upon completion, they provided subjective ratings for the valence and arousal levels of their emotional identification and experience with the segment. Following a 20-s rest period, they proceeded to the next phase.

During the analytical teaching phase, the teacher selects a teaching method to instruct students. The specific teaching design includes instructional prompts and content, lasting approximately 4 min (see Figure 1c). Partial learning (breaking music into segments for sequential study) generates more interaction and yields better learning outcomes than holistic learning (studying a piece of music in its entirety) [37]. Therefore, teachers decomposed each piece into four segments for instruction. The Visual Mental Imagery Group employed the teacher’s verbal guidance as the core medium, guiding students to experience music by associating verbally described scenes with the heard music. The teacher first verbally described a scene strictly matching the target musical piece, prompting students to engage in internal mental imagery exercises, followed by the presentation of the musical piece. Specific instructional content was determined prior to class through discussions with the teacher group based on the music’s background and characteristics (see Supplementary Material S1 and S2). The AR group employed AR visual scenes as the core medium (consistent with the verbally described scenes, differing only in presentation format). Teachers guided students to associate the AR-viewed scenes with the heard music to experience it. Teachers used an iPad to display a pre-recorded video strictly matching the target musical piece within the AR glasses worn by students, facilitating externally guided mental imagery exercises. Subsequently, the musical piece was presented. Throughout the process, teachers did not wear AR glasses but controlled the AR video playback via iPad while supervising the entire procedure. Teachers maintained neutral facial expressions, consistent volume, and a calm tone throughout instruction. The no-instruction control group received no imagery guidance, teachers and students merely listened to the same music segment synchronously. After a 20-s break, the next phase commenced.

During the comprehensive listening phase, students were guided to apply the knowledge or methods acquired during the analytical instruction phase to revisit the musical pieces. Following this, they completed subjective assessments of the emotional valence and arousal level associated with identifying and experiencing the mood of each segment. Additionally, they solved four math problems as distracting stimuli. Throughout the process, the teacher’s presence was minimized to avoid influencing students’ button presses. Teachers focus entirely on the teaching process itself, refraining from intervening or observing students’ specific reactions. Assessment equipment (keyboards and screens) is positioned directly in front of students. The entire experiment lasted approximately 40 min, involving the learning of four musical pieces (two positive, two negative) averaging 120 s each. Musical order was balanced across participants. Both teachers and student participants received compensation upon completion.

### 2.4. Near-Infrared Data Acquisition

**Figure 2:**
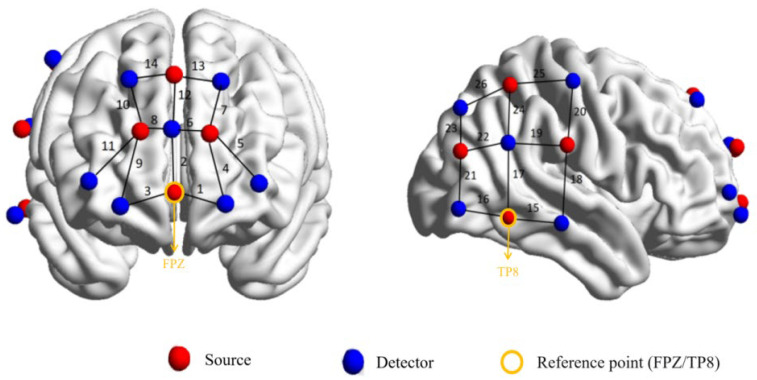
Optical Probe Arrays and Channels on a 3D Brain. Note: Red indicates the emitter electrode, blue indicates the receiver electrode, and numbers denote channels.

This study employed the NIRScout 16–24 desktop near-infrared functional brain imaging system (NIRx Medizintechnik GmbH, Berlin, Germany) to acquire brain functional signals. The system operated at a sampling frequency of 7.8125 Hz, utilizing near-infrared light at wavelengths of 760 nm and 850 nm to monitor real-time changes in the concentrations of oxygenated hemoglobin (HbO) and deoxygenated hemoglobin (HbR) within the cerebral cortex. Given the prefrontal cortex’s critical role in emotional processing [38] and the right temporo-parietal junction’s key function in social interaction learning [22], these two brain regions were designated as regions of interest (ROIs) (see Table 1). The optical electrode array comprised 8 emitters and 12 detectors, forming 26 effective channels per learner-teacher pair (channel layout shown in Figure 2). Electrodes were arranged in an interleaved pattern with 3 cm spacing between adjacent light sources and detectors. The probe array’s central axis aligned with the sagittal reference plane according to the international 10–20 system, with the center of the lowest row of optical nodes positioned at the Fpz reference point. The frontal lobe region features 4 emitters and 7 detectors, forming 14 measurement channels; the right temporo-parietal junction area has 4 emitters and 5 detectors, comprising 12 channels. All probe positions were standardized across participants and calibrated prior to the experiment. Spatial localization of channels was obtained using a 3D virtual positioning system (FASTRAK, Polhemus, Colchester, VT, USA). Channel coordinates were then transformed into MNI standard space using the spatial registration module within the NIRS-SPM toolbox. Detailed coordinate data are provided in Supplementary Material S2.

### 2.5. Data Analysis

#### 2.5.1. Subjective Evaluation Data Analysis

Statistical analysis of students’ subjective rating data was conducted using SPSS 26.0 software. All dependent variables generally met assumptions of normality and homogeneity of variance (see Supplementary Material S4.1). First, a three-factor repeated measures ANOVA was conducted with music emotion recognition (valence, arousal) and music emotion experience (valence, arousal) from the subjective ratings as dependent variables. The design was 3 (teaching method: control group, visual mental imagery group, AR teaching group) × 2 (music emotion valence: positive, negative) × 2 (time: pre-teaching, post-teaching). This explored differences in music emotion processing before and after instruction across teaching methods.

#### 2.5.2. Near-Infrared Data Processing

Preprocessing: Acquired signals were imported into the nirsLAB analysis pipeline. The signal-to-noise ratio (SNR) for each channel was assessed based on the coefficient of variation (CV) metric. Channels exhibiting a CV exceeding 15% at both wavelengths were excluded [39,40,41]. Subsequently, further processing was performed using the Matlab R2013b platform and the Homer2 toolkit [42], comprising the following steps: (1) Converting raw optical density signals into oxygenated hemoglobin (HbO_2_) and deoxygenated hemoglobin (HbR_2_) concentrations; (2) Correcting global signal components using principal component analysis (hmrMotionCorrectPCA, nSV = 0.80); (3) Implementing a correlation-based signal enhancement algorithm (turnon = 1) to suppress motion artifacts; (4) Sequential bandpass filtering (0.01–0.08 Hz) and bandstop filtering (0.15–0.3 Hz) to preserve low-frequency neural activity while excluding physiological noise (e.g., respiratory rhythm); (5) Conversion of signals to HbO concentration changes based on the modified Beer-Lambert law, followed by baseline correction [43]. Given that HbO exhibits a higher signal-to-noise ratio, greater sensitivity to cerebral blood flow changes, and broader applicability in fNIRS studies [44,45], subsequent analyses in this study focused on HbO concentration changes.

Inter-brain synchronization analysis: The wavelet transform coherence algorithm was employed to analyze neural activity synchrony between teachers and students during instructional tasks. This method has been widely applied in hyperscanning studies of teacher–student interactions [46,47]. The analysis workflow is as follows: First, WTC values for each frequency-channel pair were calculated separately during the resting and teaching phases, then averaged over the time dimension to obtain corresponding WTC matrices. Considering that inherent neural activity similarities between individuals may influence task-related brain synchronization, this study uses resting-state brain synchronization levels as the baseline [48]. The IBS value during the teaching phase is defined as the teaching-phase coherence value minus the resting-state coherence value. The resting phase data were selected from the 120 s preceding music listening to ensure baseline stability. For frequency filtering, the study first analyzed the full frequency range of 0.027–1 Hz. To exclude high-frequency noise interference from cardiac pulsations (0.8–2.5 Hz) [45] and respiratory activity (0.20–0.30 Hz) [49], while referencing previous teacher–student interaction studies indicating IBS enhancement predominantly occurs above 0.025 Hz [46]. The frequency range of 0.036–0.2 Hz (corresponding to periods of 5–28 s) was ultimately selected as the region of interest. All IBS data underwent Fisher-z transformation for distribution normalization prior to statistical analysis. Subsequently, channel averages were calculated for regions of interest across conditions. Repeated-measures ANOVA was employed to examine the effects of teaching methods (visual mental imagery group, AR teaching group, control group) and musical affect valence (positive, negative) on inter-brain synchronization during teaching phases. Multiple comparisons were corrected using the false discovery rate (FDR) method (significance level set at *p* < 0.05). Finally, channel-level statistical results were visualized using the BrainNet Viewer tool [50].

## 3. Results

A one-way ANOVA was performed to examine potential baseline differences in demographic characteristics and music emotion processing abilities among the three groups prior to the intervention. The analysis showed no statistically significant effects for any of the assessed variables (*F*(2, 78) ≤ 2.61, *p* ≥ 0.08, *η_p_*^2^ ≤ 0.06; refer to Supplementary Material S4.2), demonstrating the effectiveness of the random assignment procedure in creating equivalent groups across experimental conditions.

### 3.1. Manipulation Check of the Teaching Intervention

To examine whether the two teaching interventions successfully engaged their targeted mental process—visual mental imagery—we conducted separate 2 (Time: pre-test, post-test) × 3 (Group: AR instruction, visual imagery instruction, no-instruction control) repeated-measures analyses of variance on both the prevalence and vividness of visual imagery. The analyses revealed non-significant three-way interactions (Music Valence × Time × Teaching Method) for both measures (all *ps* > 0.05), indicating that the effect of the teaching method was consistent across music with different emotional valences. Furthermore, the results revealed significant teaching effects that exhibited a method-specific pattern.

The analysis of visual imagery prevalence scores revealed a significant Time × Teaching Method interaction (*F*(2, 78) = 49.66, *p* < 0.001, *η_p_*^2^ = 0.56). Simple effect analysis indicated no between-group differences at pre-teaching (*F* = 0.55, *p* = 0.58), confirming equivalent baseline levels. At post-test, a significant main effect of teaching method was observed (*F*(2, 78) = 48.24, *p* < 0.001). Post-teaching comparisons showed that the AR instruction group reported significantly higher visual imagery prevalence than both the visual imagery instruction group (*p* = 0.003) and the no-instruction control group (*p* < 0.001). The visual imagery instruction group also scored significantly higher than the control group (*p* < 0.001).

For visual imagery vividness, a significant Time × Group interaction was also found (*F*(2, 78) = 29.7, *p* < 0.001, *η_p_*^2^ = 0.43). Simple effect analysis confirmed no baseline differences among the three groups at pre-teaching (*F* = 1.24, *p* = 0.30). At post-test, a significant main effect of teaching method emerged (*F*(2, 78) = 40.89, *p* < 0.001). Post-teaching tests indicated that the AR instruction group reported significantly higher imagery vividness than the control group (*p* < 0.001). The visual imagery instruction group also demonstrated significantly higher vividness than the control group (*p* < 0.001). Although the AR instruction group showed a higher mean vividness score than the visual imagery instruction group, this difference did not reach statistical significance (*p* = 0.057).

The above results confirm the validity of the experimental manipulation. Both teaching methods successfully enhanced students’ visual mental imagery levels, though their mechanisms differed. Specifically, In enhancing the “prevalence” of mental imagery, the AR teaching group demonstrated a clear advantage. This indicates that by providing stable, consistent external visual anchors, AR instruction can more reliably trigger music-visual cross-modal associations. Regarding enhancing the “ vividness” of mental imagery, both teaching methods were effective in elevating it to a high level. This indicates that once visual mental imagery is successfully triggered—whether through external guidance or internal generation—learners can construct detailed mental images.

### 3.2. Group Differences in Musical Emotion Processing

**Figure 3:**
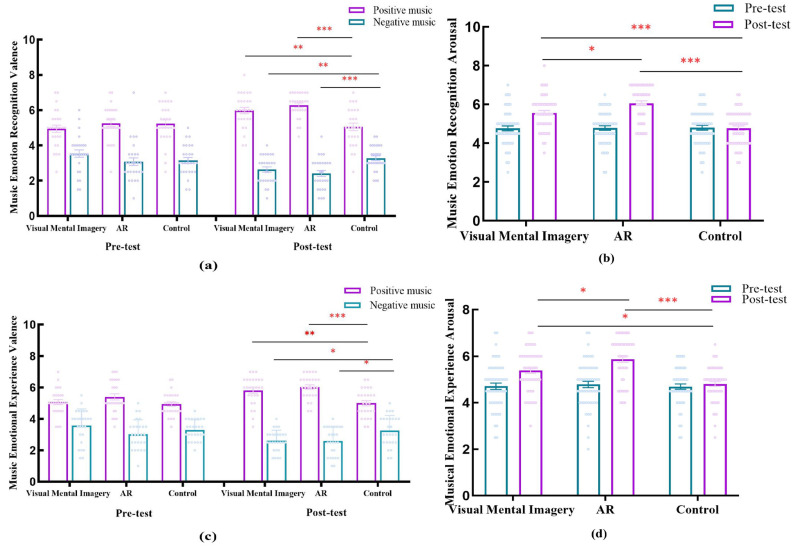
Music emotion processing results across three groups (music emotion recognition valence (**a**), music emotion recognition arousal (**b**), music emotion experience valence (**c**), music emotion experience arousal (**d**)). Note: * *p* < 0.05, ** *p* < 0.01, *** *p* < 0.001.

We sought to examine whether different teaching methods influence students’ musical emotion processing. Using a mixed-design ANOVA with time (pre-teaching, post-teaching) and musical valence (positive, negative) as within-subjects factors, and teaching method (no instruction, visual mental imagery instruction, AR instruction) as between-subjects factors, we investigated students’ musical emotion recognition and musical emotion experience (valence and arousal) separately. Table 2 presents the means and standard deviations for music emotion recognition and emotion experience (valence and arousal), while Figure 3 provides a visual representation of the same data. A three-factor repeated measures ANOVA was conducted on the music emotion processing scores across the three groups, yielding the following results:

Regarding affective valence in emotion recognition, a significant three-way interaction was found, *F*(2, 78) = 20.33, *p* < 0.001, *η_p_*^2^ = 0.34(see Figure 3a). Simple effects analysis was further conducted using Bonferroni correction. Results indicated no significant differences in pretest valence across the three groups under the positive music condition (*p* = 0.47). Posttest results revealed that the AR group (6.28 ± 0.18) scored significantly higher than the control group (5.06 ± 0.18; *p* < 0.001), while the visual mental imagery group (5.98 ± 0.18) significantly exceeded the control group (*p* = 0.001). No significant difference existed between the AR instruction and visual mental imagery groups (*p* = 0.73). Intra-group pre-post comparisons revealed that the post-test scores of the AR group (6.28 ± 0.18) were significantly higher than the pre-test scores (5.26 ± 0.20; *p* < 0.001). The post-test affective scores of the visual mental imagery group (5.98 ± 0.18) were significantly higher than the pre-test scores (4.94 ± 0.20; *p* < 0.001). However, no significant difference was found between pre- and post-tests in the control group (pre-test: 5.24 ± 0.20; post-test: 5.06 ± 0.17; *p* = 0.35). Under the negative music condition, no significant differences existed in pretest affect scores across the three groups (*p* = 0.22). Posttest results showed that the AR teaching group (2.41 ± 0.15) scored significantly lower than the control group (3.28 ± 0.15; *p* < 0.001), while the visual mental imagery group (2.63 ± 0.15) scored significantly lower than the control group (*p* = 0.012). No significant difference existed between the AR instruction group and the visual mental imagery group (*p* = 0.93). Intra-group pre-post comparisons revealed that the post-test effect size in the AR group (2.41 ± 0.15) was significantly lower than the pre-test (3.07 ± 0.2; *p* < 0.001). The post-test affective scores of the visual mental imagery group (2.63 ± 0.15) were significantly lower than the pre-test scores (3.54 ± 0.20; *p* < 0.001). In contrast, no significant difference was found between pre- and post-tests in the control group (pre-test: 3.15 ± 0.20; post-test: 3.28 ± 0.15; *p* = 0.45).

Regarding emotional recognition arousal. A significant interaction between time and teaching method was observed, *F*(2, 78) = 28.31, *p* < 0.001, *η_p_*^2^ = 0.42 (see Figure 3b). Simple effects analysis revealed no pretest differences among the three groups (*p* = 0.98). Posttest emotional recognition arousal scores showed that the AR group (6.07 ± 0.13) was significantly higher than the control group (4.77 ± 0.13; *p* < 0.001) and the visual mental imagery group (5.57 ± 0.13; *p* = 0.029). The visual mental imagery group scored significantly higher than the control group (*p* < 0.001). Furthermore, in their respective pre-post comparisons, the post-test emotional recognition arousal level of the AR teaching group (6.07 ± 0.13) was significantly higher than the pre-test (4.78 ± 0.13) (*p* < 0.001), while the posttest (5.57 ± 0.13) of the visual mental imagery group was significantly higher than the pretest (4.76 ± 0.13; *p* < 0.001). In contrast, the posttest (4.77 ± 0.13) of the control group showed no significant difference from the pretest (4.80 ± 0.13; *p* = 0.83).

Regarding affective valence in emotional experiences, a significant three-way interaction was found, *F*(2, 78) = 11.65, *p* < 0.001, *η_p_*^2^ = 0.23 (see Figure 3c). Simple effects analysis with Bonferroni correction was subsequently conducted. Results indicated no significant differences in pretest valence across the three groups under the positive music condition (*p* = 0.14). Post-test results showed that the AR group (6.04 ± 0.16) scored significantly higher than the control group (5.00 ± 0.16; *p* < 0.001), while the visual mental imagery group (5.82 ± 0.16) scored significantly higher than the control group (*p* = 0.002). The difference between the AR group and the visual mental imagery group was not significant (*p* = 0.10). In the within-group pre-post comparison, the post-test efficacy score (6.04 ± 0.16) of the AR group was significantly higher than the pretest score (5.40 ± 0.17; *p* < 0.001). The post-test efficacy score for the visual mental imagery group (5.82 ± 0.16) was significantly higher than the pretest score (5.07 ± 0.17; *p* < 0.001). In contrast, no significant difference was observed between pretest and post-test scores for the control group (pretest: 4.94 ± 0.17; post-test: 5.00 ± 0.16; *p* = 0.74). Under the negative music condition, no significant difference existed in pretest affect scores across the three groups (*p* = 0.08). Post-test results showed that the AR group (2.59 ± 0.16) scored significantly lower than the control group (3.26 ± 0.16; *p* = 0.016), while the visual mental imagery group (2.61 ± 0.16) scored significantly lower than the control group (*p* = 0.02). The difference between the AR group and the visual mental imagery group was not significant (*p* = 0.1). Intra-group pre-post comparisons revealed that the post-test affective valence of the AR group (2.59 ± 0.16) was significantly lower than the pre-test (3.02 ± 0.17; *p* = 0.009). The post-test affective valence of the visual mental imagery group (2.61 ± 0.16) was significantly lower than the pre-test (3.57 ± 0.17; *p* < 0.001), while the control group showed no significant difference between pre- and post-tests (pre-test: 3.30 ± 0.17; post-test: 3.26± 0.16; *p* = 0.82).

Regarding arousal levels in emotional experiences, a significant interaction between time and teaching method was observed, *F*(2, 78) = 13.70, *p* < 0.001, *η_p_*^2^ = 0.26(see Figure 3d). Simple effects analysis revealed no pretest differences among the three groups (*p* = 0.87). Post-test emotional arousal levels showed the AR group (5.87 ± 0.14) was significantly higher than the control group (4.81 ± 0.14; *p* < 0.001) and the visual mental imagery group (5.39 ± 0.14; *p* = 0.046). The visual mental imagery group was significantly higher than the control group (*p* = 0.011). In respective pre-post comparisons, the post-test emotional arousal level of the AR teaching group (5.87 ± 0.14) was significantly higher than the pre-test (4.79 ± 0.13; *p* < 0.001), while the post-test emotional arousal level in the visual mental imagery group (5.39 ± 0.14) was significantly higher than the pretest (4.71 ± 0.13; *p* < 0.001). The post-test emotional arousal level in the control group (4.81 ± 0.14) showed no significant difference from the pretest (4.69 ± 0.13; *p* = 0.40).

### 3.3. Group Differences in IBS

**Figure 4:**
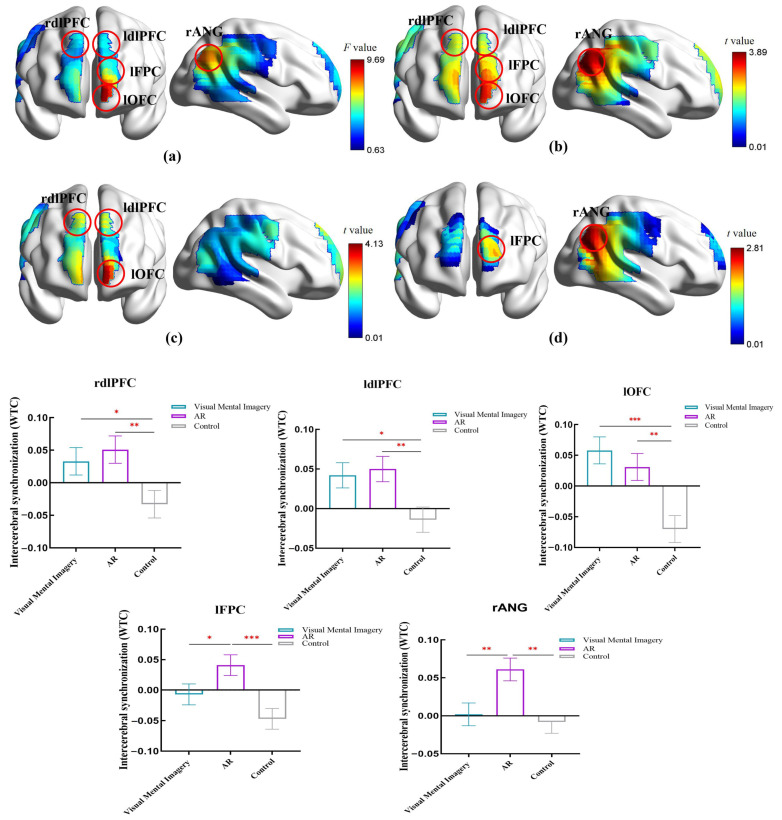
Results of analysis of variance. (**a**) shows the main effect of teaching method. (**b**–**d**) present the results of pairwise comparisons. (**b**) In brain regions (lFPC, lOFC, ldLPFC, rdlPFC, rANG), significant differences existed between the AR teaching group and the control group in IBS during the teaching phase. (**c**) Significant differences in IBS during the teaching phase were observed between the visual mental imagery group and the control group in brain regions (lOFC, ldLPFC, rdLPFC). (**d**) Significant differences in IBS during the teaching phase were observed between the AR teaching group and the visual mental imagery group in brain regions (lFPC, rANG). Note: * *p* < 0.05, ** *p* < 0.01, *** *p* < 0.001.

We calculated the mean IBS values for the three groups across all experimental conditions within the 0.036–0.2 Hz frequency band. This measurement was derived from the difference between the “Teaching stage (Figure 1b)” and the “Rest1 baseline (Figure 1b)” in the regions of interest (ROIs). Using the mean IBS values of the ROI brain regions across the three groups (see Supplementary Material Table S5) as the dependent variable, we conducted a two-factor repeated measures ANOVA (2 (Music Emotional Valence: Positive, Negative) × 3 (Control Group, AR Instruction Group, Visual Mental Imagery Instruction Group)). Multiple comparisons were corrected using the FDR method (*p* < 0.05). The ANOVA results and pairwise comparison near-infrared brain imaging maps are presented in Figure 4, with findings as follows.

In the lFPC, the main effect of teaching method was significant, *F*(2, 78) = 6.75, *p*_FDR_ = 0.014, *η_p_*^2^ = 0.15. The AR teaching group (0.041 ± 0.017) showed significantly higher IBS than the control group (−0.047 ± 0.017; *p* < 0.001) and the visual mental imagery teaching group (−0.01 ± 0.017; *p* = 0.037). The difference between the visual mental imagery teaching group and the control group was not significant (*p* = 0.13).

In the lOFC, the main effect of teaching method was significant, *F*(2, 78) = 9.65, *p*_FDR_ < 0.001, *η_p_*^2^ = 0.2. The AR group (0.031 ± 0.022) showed significantly higher IBS than the control group (−0.007 ± 0.022; *p* = 0.002). The visual mental imagery teaching group (0.058 ± 0.022) showed significantly higher IBS than the control group (*p* < 0.001). The difference in IBS between the AR teaching group and the visual mental imagery teaching group was not significant (*p* = 0.38).

In the ldlPFC, the main effect of teaching method was significant, *F*(2, 78) = 4.91, *p*_FDR_ = 0.035, *η_p_*^2^ = 0.11. The AR teaching group (0.05 ± 0.016) showed significantly higher IBS than the control group (−0.014 ± 0.016; *p* = 0.005). The visual mental imagery teaching group (.042 ± 0.016) showed significantly higher IBS than the control group (*p* = 0.014). The difference in IBS between the AR teaching group and the visual mental imagery teaching group was not significant (*p* = 0.73).

In the rdlPFC, the main effect of teaching method was significant, *F*(2, 78) = 4.45, *p*_FDR_ = 0.042, *η_p_*^2^ = 0.1. The AR group (0.051 ± 0.021) showed significantly higher IBS than the control group (−0.033 ± 0.021; *p* = 0.006). The visual mental imagery teaching group (0.033 ± 0.021) also showed significantly higher IBS than the control group (*p* = 0.029). The difference in IBS between the AR teaching group and the visual mental imagery teaching group was not significant (*p* = 0.54).

In the rANG, the main effect of teaching method was significant, *F*(2, 78) = 5.93, *p*_FDR_ = 0.028, *η_p_*^2^ = 0.13. The AR group (0.061 ± 0.015) showed significantly higher IBS than the control group (−0.008 ± 0.015; *p* = 0.002) and the visual mental imagery teaching group (0.015 ± 0.015; *p* = 0.008). The difference in IBS between the visual mental imagery group and the control group was not significant (*p* = 0.66).

In summary, a significant main effect of teaching method was found for the IBS values in all five ROIs. Crucially, this effect demonstrated a clear hierarchical pattern. For the lFPC and rANG, pairwise comparison shows that the AR instruction group showed significantly higher IBS than both the visual imagery instruction group and the no-instruction control group, while the latter two groups did not differ significantly from each other. For the three regions of lOFC, ldlPFC, and rdlPFC, pairwise comparison shows that both the AR instruction group and the visual imagery instruction group exhibited significantly higher IBS than the no-instruction control group, with no significant difference between these two teaching groups.

### 3.4. Behavior–Brain Correlation Analysis

Behavioral results indicate that the AR instruction group demonstrated specific enhancements in arousal levels for both emotion recognition and emotion experience. Therefore, our brain-behavior correlation analysis aimed to examine whether the synchrony in the specifically enhanced brain regions (lFPC and rANG) within this group correlated with these behaviorally specific indicators. For the core analysis, we calculated cross-trial (positive and negative) averages for each participant across behavioral (emotion recognition arousal, emotion experience arousal) and brain activity (lFPC_IBS, rANG_IBS) measures to capture overall effects. Subsequently, we computed Pearson correlation coefficients between these two brain region metrics and the two behavioral metrics within the AR instruction group. This constituted a set of four tests, all of which underwent FDR correction. Next, as exploratory analyses, we examined the aforementioned relationships within the control group and the visual mental imagery group, and investigated the relationship between inter-subject brain synchrony and arousal levels for emotion recognition and emotion experience across all groups. All *p*-values for exploratory analyses underwent FDR correction.

**Figure 5:**
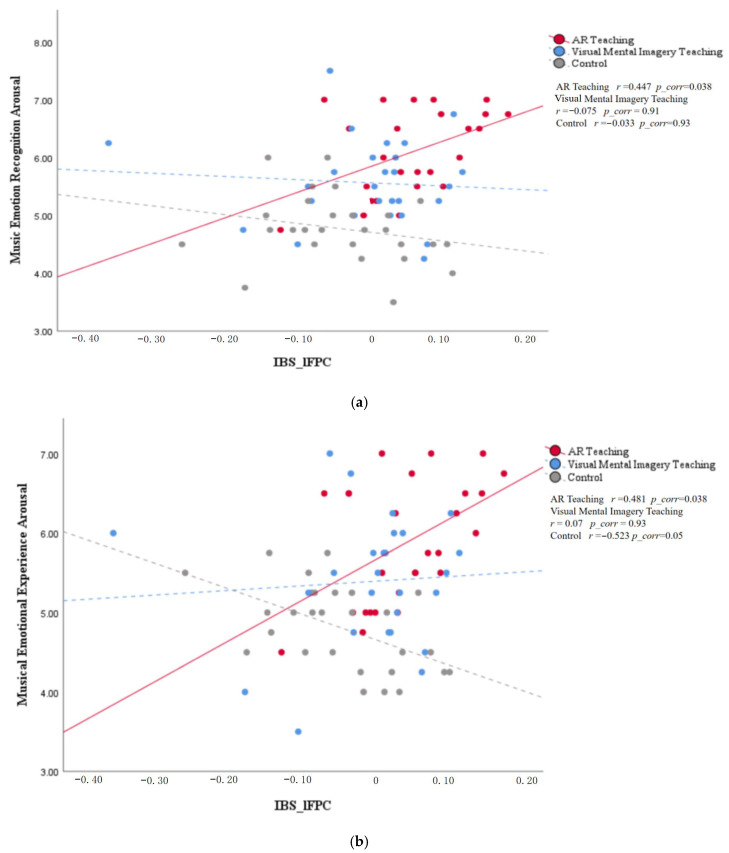
Scatter plots showing the correlation between inter-brain synchrony and emotional processing arousal levels. (**a**,**b**) present scatter plots and fitted curves illustrating changes in inter-brain synchrony within the lFPC region relative to emotional recognition arousal and emotional experience arousal across different groups. Red data points and trend lines indicate a significant moderate positive correlation in the AR instruction group. Blue and gray data points represent the visual mental imagery instruction group and the control group, respectively, with trend lines showing no statistical significance.

Correlation analysis revealed that within the AR teaching group, inter-subject synchrony in lFPC showed a significant positive correlation with emotional recognition arousal (r = 0.447, pFDR = 0.038) (see Figure 5a). Simultaneously, inter-brain synchrony in the lFPC also showed a significant positive correlation with emotional experience arousal (r = 0.481, pFDR = 0.038) (see Figure 5b). The remaining correlations did not reach significance (all *p* > 0.05). These findings indicate that inter-brain synchrony in the left anterior cingulate cortex during the teaching phase specifically predicted the arousal intensity of music–emotion processing in subsequent AR teaching group individuals. However, exploratory analysis revealed a marginal negative correlation between music-induced emotional experience arousal and lFPC IBS in the control group (r = −0.523, pFDR = 0.05), while no other correlations reached statistical significance (all *p* > 0.05). Notably, the negative association between lFPC synchronization and experiential arousal observed in the control group stands in sharp contrast to the positive correlation identified in the AR instruction group. This divergence suggests that identical neural activity may serve context-dependent functional roles, with the presence or absence of structured teaching intervention fundamentally altering its relationship to behavioral outcomes. Furthermore, the absence of significant correlations between rANG synchronization and behavioral measures across all groups indicates that lFPC may represent a specific neural signature of teaching intervention effects.

### 3.5. The Mediating Role of IBS Between Teaching Strategies and Emotional Processing Arousal

To examine the mediating role of inter-brain synchrony in the lFPC region on the effect of AR instruction on emotional processing arousal, we employed the PROCESS macro (version 4.0, model 4) developed for mediation analysis [51]. Bootstrap sampling (5000 iterations) was employed to examine the mediating effect of lFPC IBS between teaching method (AR group vs. control group) and emotional arousal. Two independent mediation models were established, using emotional recognition arousal and emotional experience arousal as dependent variables, respectively.

**Figure 6:**
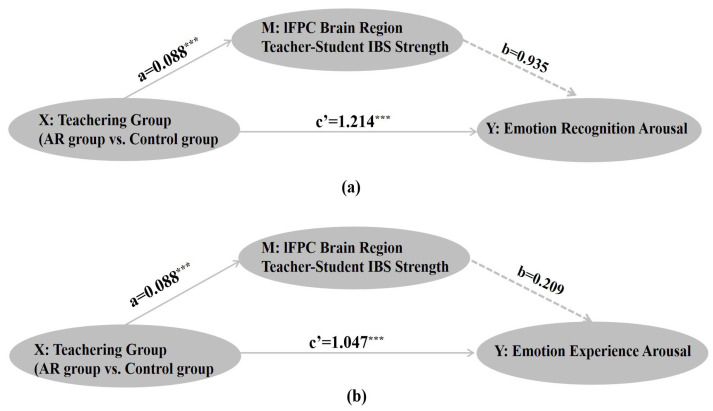
Mediating model of lFPC interbrain synchrony in the relationship between teaching methods and emotional arousal levels, with emotional recognition arousal (**a**) and emotional experience arousal (**b**) as dependent variables. Although both mediating models predicted higher lFPC IBS in the AR teaching group (compared to the control group) (path a), no significant mediating effects were found. *** *p* < 0.001.

In the model with emotion recognition arousal as the dependent variable, the overall effect analysis revealed that teaching method exerted a significant positive influence on emotion recognition arousal (B = 1.3, SE = 0.18, t = 7.09, *p* < 0.001). Path analysis revealed that teaching method significantly predicted lFPC inter-subject synchrony (path a: B = 0.09, SE = 0.02, t = 3.93, *p* < 0.001). However, controlling for teaching method, the predictive effect of lFPC inter-brain synchrony on emotion recognition arousal was no longer significant (path b: B = 0.94, SE = 1.14, t = 0.82, *p* = 0.42). The direct effect of teaching method on emotion recognition arousal remained significant (Path c’: B = 1.21, SE = 0.21, t = 5.82, *p* < 0.001). Indirect effect analysis revealed that the mediation effect value via lFPC inter-brain synchrony was 0.082, with a 95% Bootstrap confidence interval of [−0.117, 0.330] that included zero, indicating this mediation effect was not significant (see Figure 6a).

In the model with emotional experience arousal as the dependent variable, we observed a highly consistent pattern of results. The overall effect of teaching method on emotional experience arousal was significant (B = 1.07, SE = 0.18, t = 5.89, *p* < 0.001). Teaching method also significantly predicted lFPC inter-brain synchrony (path a: B = 0.09, SE = 0.02, t = 3.93, *p* < 0.001), but lFPC inter-subject synchrony did not significantly predict emotional experience arousal (path b: B = 0.21, SE = 1.14, t = 0.18, *p* = 0.86). The direct effect of teaching method was also significant (path c’: B = 1.05, SE = 0.21, t = 5.03, *p* < 0.001). The indirect effect value was 0.018, with a 95% Bootstrap confidence interval of [−0.193, 0.270] that similarly included zero, again confirming the absence of a mediating effect (see Figure 6b).

Collectively, these results suggest that lFPC inter-brain synchrony, while sensitive to the AR teaching intervention, does not function as a significant mediator for enhanced emotional arousal. This dissociation suggests that lFPC synchrony may be a correlate rather than a causal mechanism of the pedagogical benefits, pointing to the involvement of other, as yet unidentified, pathways.

## 4. Discussion

### 4.1. Key Findings

This study is the first to investigate the effects of different teaching methods guiding visual mental imagery on listeners’ musical emotion processing within a music education context. Through a combined behavioral measurement and fNIRS hyperscanning technique, the study constructed authentic teaching scenarios in a laboratory setting. Data were collected from 3 teacher–student pairs and 81 students, comparing the effects of three conditions on musical emotion processing: externally guided integration (AR group), internally generated simulation (visual mental imagery group), and no instruction (control group). Key findings are as follows.

First, the behavioral results indicate that both teaching methods designed to activate visual mental imagery mechanisms were effective in enhancing affective valence. This is reflected in the post-tests of both groups, where participants rated positive music more favorably and negative music more negatively. This finding indicates that both teaching methods enhanced students’ ability to distinguish musical emotions. This aligns with the predictions of affective construction theory, which posits that providing structured contextual cues—whether internally generated or externally AR-guided—facilitates the formation of more precise emotional concepts, thereby enabling more differentiated judgments of emotional stimuli [52]. However, in terms of enhancing emotional arousal, the AR teaching group demonstrated a distinct advantage over the visual imagery teaching group. This may stem from the higher prevalence of visual mental imagery within the AR group (see Section 3.1 for operational verification results).

Neural findings revealed that both teaching methods jointly enhanced inter-subject brain synchrony in regions associated with emotional processing (lOFC) and higher-order cognitive control (bilateral dlPFC), constituting a shared neural basis for effective emotional teaching interactions. AR instruction specifically amplified neural coupling in areas linked to social cognition (lFPC) and multisensory integration (rANG). Further brain-behavior correlation analysis revealed a specific positive correlation between inter-subject synchrony in the lFPC and emotional arousal levels within the AR group, although exploratory mediation analyses did not support a causal mediating role.

In summary, this study systematically compared two teaching methods guiding visual mental imagery through behavioral measurement and fNIRS hyperscanning technology, revealing behavioral and neural differences in their effects on students’ musical emotion processing. The study not only validated the effectiveness of both approaches but also highlighted the unique advantages of AR instruction in enhancing emotional arousal and promoting neural coordination in specific brain regions. These findings empirically support the pivotal role of visual mental imagery in musical emotion processing and provide scientific grounds for future neuro-mechanism-based instructional design and optimization.

### 4.2. Theoretical Implications

This study offers a new theoretical perspective for understanding music emotion education by comparing different pathways of visual mental imagery induction: externally guided (AR group) versus internally generated (visual mental imagery group).

At the behavioral level, the two approaches shape distinct visual mental imagery. AR instruction, functioning as an efficient “cognitive scaffold,” derives its core advantage from providing a unified external visual template. This significantly enhances the universality and stability of imagery generation [15], reduces reliance on individual internal resources, and fosters reproducible imagery characterized by “high trigger rates and vividness.” Its superior emotional arousal advantage may stem from the superadditive effect of audiovisual multisensory integration, which effectively amplifies emotional responses by providing consistent cross-modal cues [15]. In contrast, while internally generated instruction is less stable than AR instruction in terms of the universality of image triggering (i.e., consistent success in every instance), it successfully activates learners’ internal resources, generating “self-driven, highly vivid” generative imagery. This demonstrates the high-quality cognitive construction potential inherent in learners’ internal generation [10]. Each approach emphasizes distinct significance: AR instruction establishes a reliable foundation of understanding for all learners, while traditional verbal guidance instruction focuses on deepening intrinsic generative and integrative capabilities.

At the neural level, the findings support and deepen the aforementioned distinction, proposing a “dual-network” neural collaboration model. First, both effective teaching approaches enhanced inter-brain synchrony within the “cognitive-emotional integration network” involving the left orbitofrontal cortex (lOFC) and dorsolateral prefrontal cortex (dlPFC). This signifies the foundational process of emotional sharing and higher-order cognitive coordination during teaching interactions, providing neural evidence for the efficacy of activating visual mental imagery as a core intervention pathway [27,53].

Second, AR instruction specifically enhanced the synchrony of the “social-multimodal integration network” involving the left frontal pole cortex (lFPC) and right angular gyrus (rANG). Synchronization in the lFPC may reflect higher-order attentional coordination driven by shared visual tasks, providing an optimized neural platform for aligning social intent and emotion. As a multisensory integration hub, the rANG’s specific synchronization indicates that AR’s standardized visual scenes serve as interpretive “anchors” for teachers and students to form unified interpretations of abstract musical emotions, thereby optimizing interpersonal semantic integration and social cognition.

In summary, this study delineates a clear theoretical framework. Traditional internally generated verbal instruction effectively activates the neural basis for individual internal imagery and foundational emotional sharing. AR instruction, however, not only activates these foundational circuits but also specifically optimizes the coordination of brain regions associated with social cognition (lFPC) and cross-modal integration (rANG). This explains, from a neural mechanism perspective, the advantage of AR in enhancing the universality of emotional arousal—it provides a more reliable “emotional pathway” by optimizing the efficiency of social cognition and higher-order integration networks.

### 4.3. Educational Significance

This study’s findings provide direct implications for educational practices guiding visual mental imagery in emotional processing. The BRECVEMA model [6] indicates that visual mental imagery mechanisms are a key pathway for music-induced emotional responses. This study further demonstrates empirically that both teaching methods inducing visual mental imagery enhance students’ ability to discern the emotional valence of music. Regarding elevating emotional arousal, the AR teaching group exhibited a distinct advantage over the visual imagery teaching group. In practical teaching, AR technology is not intended to fully replace traditional verbal instruction. Its core value lies in serving as an efficient cognitive scaffold. Given its distinct advantage in enhancing the universality of mental image triggering (see Section 3.1), AR is particularly well-suited to support learners with weaker visual mental imagery abilities and a tendency toward construction difficulties. It helps these learners establish a stable starting point for visual mental imagery, thereby bridging gaps caused by individual ability differences. In contrast, for learners with strong visual mental imagery abilities and no significant construction difficulties, both teaching methods demonstrated comparable effectiveness in enhancing the vividness of mental imagery. Therefore, it is recommended to adopt a differentiated teaching approach of “assessment-adaptation” in practice. Educators may first conduct a preliminary assessment of students’ visual imagery generation abilities through simple tasks. If students demonstrate significant difficulties, AR technology can be introduced as an introductory tool and cognitive scaffolding. If students possess adequate abilities, both methods remain applicable. If the core instructional goal focuses on deepening students’ intrinsic imagery construction and emotional connections, verbal guidance should be prioritized. This approach enables teaching interventions tailored to students’ cognitive characteristics.

Additionally, the neural indicators examined in this study—such as inter-brain synchrony—serve as a window for researchers to understand the neurological foundations of teaching interactions and provide metrics for evaluating the overall effectiveness of affective instructional design. For frontline educators, the practical focus should remain on designing and selecting high-quality instructional content (e.g., the quality of AR scenarios, the precision of verbal guidance) and observing student states.

### 4.4. Limitations and Future Directions

Although this study has yielded several innovative findings, it also has certain limitations, which point to feasible directions for future in-depth research.

Methodologically, first, the fNIRS technique employed in this study primarily covered the prefrontal cortex, failing to comprehensively monitor other brain regions associated with musical emotion processing, such as the temporal and parietal Lobes [54]. Future research could integrate techniques like EEG (high temporal resolution) or fMRI (high spatial resolution) to provide a more comprehensive observation of brain activity. Second, despite preprocessing to filter high-frequency noise, analyses primarily based on HbO signals struggle to fully exclude the influence of extremely low-frequency systemic physiological fluctuations. Future work should incorporate HbR signal analysis and concurrently record physiological indicators like ECG and respiration to more rigorously separate neural activity from systemic physiological contributions. Third, the current approach employs structured visual guidance; future studies may utilize fully functional AR systems to investigate the effects of varying levels of virtual-physical interaction. Fourth, the limited number of instructors (*n* = 3) may introduce residual confounding effects from teaching styles. Future research should expand the instructor sample size and employ multilevel models to control for individual differences. Finally, it is difficult to completely rule out the persistent influence of the novelty of the AR devices themselves on arousal levels. Future studies may adopt longitudinal designs or establish novelty control groups to isolate this factor.

Regarding measurement tools. First, this study employed a valence-arousal two-dimensional model to capture core emotional characteristics but failed to depict specific discrete emotions. Introducing tools such as the Geneva Emotional Music Scale [33] could enable more refined measurement of specific emotions evoked by music. Additionally, current measurements primarily target phased overall responses. Future studies may employ continuous measurement techniques like dynamic scoring to more sensitively capture real-time fluctuations and subtle shifts in emotional responses during instruction. Third, the “delayed integration” design used in this study involves working memory processes. Future research could incorporate baseline measurements of working memory capacity to analyze the interaction between individual cognitive abilities and different instructional pathways.

Regarding ecological validity and long-term effects. First, the non-significant mediation effect highlights a limitation of this study. Measurement precision, sample size, and control for potential confounding variables (such as the novelty effect of AR) may all influence the detection power of complex mediation models [55]. Future research could further clarify the precise role neural synchrony may play in teaching effectiveness through larger samples, multi-time-point measurements, and more refined experimental designs. Furthermore, this study primarily focuses on immediate effects. Investigating whether AR-guided external cues combined with internally generated visual mental imagery instruction can produce enduring visual mental imagery and neuroplasticity changes represents a crucial next step in evaluating its educational value.

## 5. Conclusions

This study employed fNIRS hyperscanning technology to compare neural and behavioral differences between externally guided augmented reality (AR) instruction and internally generated simulation in guiding students’ visual mental imagery and influencing their musical affect processing. Key findings are as follows: At the behavioral level, both instructional methods effectively enhanced students’ recognition of musical affect valence, while AR instruction demonstrated unique advantages in enhancing affect arousal. At the neural level, both methods shared activation in brain regions involved in affective processing (lOFC) and imaginative reasoning (bilateral dlPFC). AR instruction additionally specifically enhanced activity in regions associated with social cognition (lFPC) and multimodal integration (rANG). Further correlation analysis revealed a significant positive correlation between inter-subject synchrony in the lFPC and emotional arousal within the AR group. This neural indicator may serve as a marker for AR-induced high-arousal experiences, though its causal mechanisms require further validation. This study confirms visual mental imagery as a key mechanism for musical emotion processing at both behavioral and neural levels, providing empirical support for developing personalized music emotion teaching strategies.

## Figures and Tables

**Table 1:** ROIs and corresponding fNIRS channels.

Brain Areas	ROI	Channel	Brain Areas	ROI	Channel
FPC	lBA10	2, 4, 5, 6	ANG	rBA39	20, 24, 25
	rBA10	8, 9, 11	SMG	rBA40	17, 18, 19
OFC	lBA11	1	ITG	rBA20	21
	rBA11	3	TPGmid	rBA21	22
dlPFC	lBA9	7, 12, 13	FFG	rBA37	23
	rBA9	10, 14	V3	rBA19	26
SI	rBA1	15	STG	rBA22	16

**Table 2:** Mean and standard deviations for the dependent measures of three groups.

Dependent Variables		Control Group (*n* = 27)								Visual Mental Imagery Teaching Group (*n* = 27)								AR Teaching Group (*n* = 27)							
		Pre-Test				Post-Test				Pre-Test				Post-Test				Pre-Test				Post-Test			
		Positive Music		Negative Music		Positive Music		Negative Music		Positive Music		Negative Music		Positive Music		Negative Music		Positive Music		Negative Music		Positive Music		Negative Music	
		M	SD	M	SD	M	SD	M	SD	M	SD	M	SD	M	SD	M	SD	M	SD	M	SD	M	SD	M	SD
Musical Emotional Processing	Emotion Recognition Valence	5.24	1.10	3.15	0.88	5.06	1.08	3.28	0.71	4.94	1.06	3.54	1.07	5.98	0.96	2.63	0.78	5.26	0.99	3.07	1.15	6.28	0.70	2.41	0.90
	Emotion Recognition Arousal	4.85	1.05	4.74	0.75	4.91	0.94	4.63	0.79	4.98	0.95	4.54	0.84	5.76	0.84	5.37	0.88	5.00	0.71	4.56	0.96	6.46	0.62	5.67	0.99
	Emotion Experience Valence	4.94	0.74	3.30	0.64	5.00	0.88	3.26	0.96	5.07	0.84	3.57	1.06	5.81	0.91	2.61	0.67	5.41	1.03	3.02	0.92	6.04	0.75	2.59	0.90
	Emotion Experience Arousal	4.83	0.75	4.56	0.93	4.93	0.77	4.69	0.77	5.00	0.94	4.43	1.00	5.65	0.84	5.13	0.93	5.06	0.84	4.52	1.09	6.31	0.62	5.43	1.08

## Data Availability

The data that support the findings of this study are openly available in “Zenodo (https://zenodo.org)” at https://doi.org/10.5281/zenodo.17918209.

## Supplementary Materials

The following supporting information can be downloaded at: https://www.mdpi.com/article/10.3390/brainsci16010066/s1, Supplementary Materials S1: Musical materials used in this study. Supplementary Materials S2: Teaching stage specific lesson plans. Supplementary Materials S3. Spatial Localization of fNIRS Channels. Supplementary Materials S4. Supplementary Materials for Behavioral Outcomes. Supplementary Materials S5. IBS Results Supplement.
