# Supplementary material for: From Imagination to Immersion: The Impact of Augmented Reality Instruction on Musical Emotion Processing: An fNIRS Hyperscanning Study
Source: Brain Sci. 2025 Dec 31;16(1):66. doi: 10.3390/brainsci16010066 (PMC12839031; doi:10.3390/brainsci16010066)
Supplement: Supplementary file 1 [file brainsci-16-00066-s001.zip › brainsci-4068547-supplementary.pdf]

Supplementary Material

S1 Musical materials used in this study

S1.1 Introduction to Pipa Music

The Pipa is a quintessential plucked string instrument in traditional Chinese music, boasting a history spanning over two millennia. Its name traces back to the Qin Dynasty (221–206 BC), originating from a long-necked, round-bodied instrument. The term "Pipa" derived from the playing technique: "pi" referred to plucking the strings forward, while "pa" meant hooking them backward. During the Northern and Southern Dynasties (420–589 AD), the crooked-neck Pipa from the Western Regions was introduced to Central China, merging with the indigenous straight-neck Pipa to form a new hybrid instrument. By the Tang Dynasty (618–907 AD), the Pipa reached its zenith, becoming widely popular in both court and folk music with refined playing techniques and standardized construction. Further improvements during the Ming (1368–1644) and Qing (1644–1912) dynasties culminated in its modern form—featuring six xiang (phase frets) and twenty-four pin (positional frets).

Contemporary Pipa performance adopts an upright posture, with the instrument held at approximately 45 degrees to the player’s body (Fig. S1). The left hand presses the strings to control pitch, while the right hand—fitted with artificial nails—produces diverse timbres through techniques like plucking , lifting , tremolo, and strumming . Characterized by its bright, crystalline tone and distinctly articulated notes, the Pipa delivers crisp, resonant attacks with expressive tension. It masterfully conveys both

delicate lyrical passages and grand dramatic scenes, solidifying its status as one of Chinese traditional music's most versatile and evocative instruments.

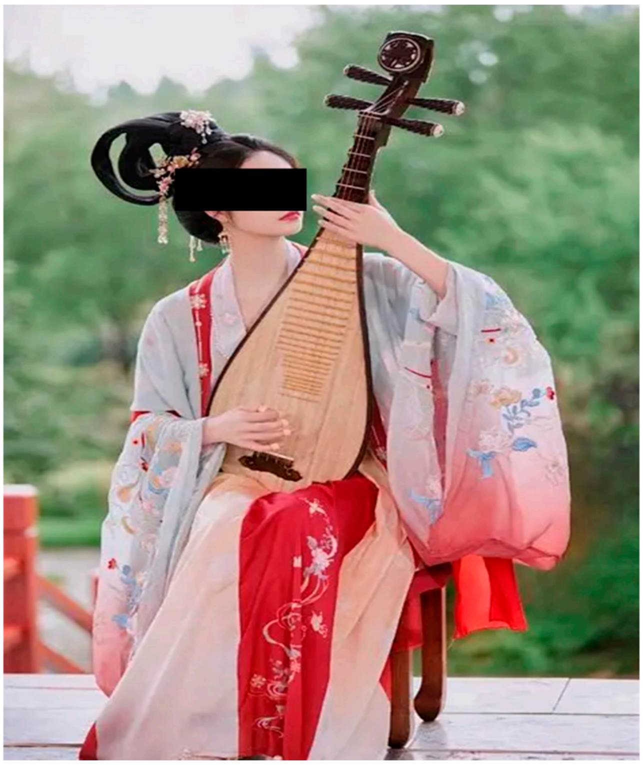

Figure S1 Examples of Pipa instrument performance

S1.2 The four musical segments used in the experiment and their creative backgrounds

a. High mountain and flowing water (Positive)

Background of Music Composition: In the Spring and Autumn period (4th century BCE), the story of Bo Ya and Zhong Ziqi was recorded in Liezi. Bo Ya was a master of the qin, while Zhong Ziqi possessed a remarkable ability to understand his music. Once, when Bo Ya played with mountains in mind, Zhong Ziqi exclaimed, "How majestic! It is like Mount Tai." When his melody turned to flowing water, Zhong Ziqi responded, "How vast! It resembles the great rivers." Another time,

caught in a storm at the foot of Mount Tai, Bo Ya began to play, first depicting rain falling in the mountains, then the surge of floods and the crash of landslides. Each time, Zhong Ziqi grasped the essence of the music perfectly. Moved, Bo Ya set aside his qin and sighed, “You truly understand what I express through music—my thoughts are an open book to you.” From then on, they became lifelong soulmates.

b. Send me a rose (Positive)

Background of Music Composition: Based on a traditional Uyghur love song, this piece employs versatile pipa techniques to vividly portray the passion and joy of young lovers. Adapted by Wang Fandi in 1961 from a Xinjiang folk song of the same name, the composition retains the melodic features of Xinjiang music while using dynamic rhythms and lively melodies to express the devotion and happiness of youth in pursuit of love.

c. Wei River Affection (Negative)

Background of Music Composition:Named after the Wei River in Shaanxi, the piece "Weishui" expresses a deep love and nostalgia for the homeland. Through techniques such as harmonics and vibrato, it mimics natural sounds like the river's ripples, the whisper of wind, and birdsong, creating a vivid scene where mountains and waters reflect one another. The melody draws on the "Kuyin" mode of Qin Opera—featuring subtle pitch variations like slightly lowered si and fa—to evoke a profound, lingering sense of homesickness through its gentle and winding phrases.

d. The king unloads his armor (Negative)

Background of Music Composition:The composition builds a profound narrative that moves from solemnity and melancholy, through struggle and tragedy, to final emptiness and solitude. It depicts not only the fury of battle but also the inner turmoil and eventual fall of a hero. It opens with low, slow, and monotonous drumbeats—less like a call to arms than a series of heavy sighs weighing on the heart. This creates not a sense of courage, but one of oppression and ominous foreboding, as though a storm is gathering before the war. The melody then turns dignified and intense, accelerating into chaos as the battle reaches its peak. Long, sorrowful phrases and expressive left-hand techniques such as pushing, pulling, and vibrato produce a weeping, choked-up effect—revealing the most vulnerable and painful fractures within a heroic heart. In the end, the music grows sparse and desolate, like survivors dispersing in silence, leaving only the vast twilight over a battlefield after the smoke has cleared.

S2 Teaching stage specific lesson plans

S2.1 *Visual mental imagery teaching method*

Instructions:

Welcome to the music appreciation class. I will guide you in connecting the characteristics of music with your own imagination to experience it. Based on the music and the scenes described by the teacher, try to create your own mental images and feel the emotions the music evokes within you.

(High Mountains and Flowing Water)

Music 1: When you hear the light and flowing sections of the music, imagine yourself leisurely strolling along a crystal-clear riverbank. [Press 9 to listen to music]

As you listen to the second segment, the imagery in your mind gradually becomes richer and more complete. Picture the river connecting with the clear blue sky, its flow as gentle and melodic as the music itself, with a few white clouds drifting across the heavens. [Press 9 to listen to music]

During the third segment, when you hear the intense strumming, picture towering mountains and witness the spectacular sight of waterfalls cascading down their peaks. [Press 9 to listen to music]

In the final segment, when the melody becomes light and cheerful, imagine

these sounds resembling the crisp chirping of birds in the forest or the pleasant clinking of water against stones in a stream. Try using the visualization techniques your teacher guided you through to experience the music in this way. [Press 9 to listen to the music]

(Send Me a Rose)

Second piece of music: When you hear the cheerful melody, imagine passionate young people dancing gracefully around a bonfire. [Press 9 to listen to music]

Second section: During the extended passage, picture the young dancers moving in fluid, slow-motion grace. When the rhythmic melody kicks in, envision the scene bursting with youthful energy. [Press 9 to listen to the music]

Third section: Pay attention to the tremolo technique in the mid-range. Imagine young people holding tambourines, singing and dancing together. [Press 9 to listen to the music]

Final section: When you hear the tremolo sound, picture young men taking small, quick steps, roses clenched between their teeth, circling their beloved women in courtship. Try using the visualization method your teacher guided you through to experience the music. [Press 9 to listen to the music]

(Wei River Affection)

The third piece blends elements of Qinqiang opera, a traditional theater form from Northwest China. Picture a rugged Northwest man recounting the beauty of his homeland. [Press 9 to listen]

In the second segment, let the melodious music transport you to the banks of the Wei River, imagining the gentle flow of its waters. [Press 9 to listen to music]

The third segment employs a double-wheel technique. Picture the scene of villagers living in peace and contentment, tending to their livelihoods and nurturing their lives. [Press 9 to listen to music]

The fourth segment gradually deepens into a muffled soundscape. Envision a man, long separated from home, weeping alone in the night, his sobs echoing with sorrow. Soon, try using the teacher's guided visualization method to experience this while listening to the music. [Press 9 to listen to music]

(The king unloads his armor)

Fourth piece of music: Imagine this composition depicts a complete battle scene. First, visualize the eve of war—the deep, rumbling drums heralding the imminent outbreak of conflict. [Press 9 to listen to music]

The second section accelerates. Picture armies marching out, tension mounting as battle approaches. [Press 9 to listen to music]

The third section employs strumming techniques to build to the piece's most intense climax. When you hear the finger-twisting strumming, imagine fierce hand-to-hand combat. When you hear the rolling finger and sweeping wheel techniques, picture the tense scene of thousands of troops in pursuit. [Press 9 to listen to music]

The final section evokes the image of Xiang Yu, surrounded by enemy forces, bidding farewell to Yu Ji before taking his own life. Try using the visualization

techniques your teacher guided you through while listening to the music. [Press 9 to listen to music]

S3 *Spatial Localization of fNIRS Channels*

Table S2 ***Spatial Localization of fNIRS Channels***

Chanels	MNI coordinates			Broodmann's areas	Percentage of
	x	y	z		ooverlap
CH01	-12	73	-4	11 - Left Orbitofrontal area	0.5
CH02	2	68	13	10 - Left Frontopolar area	1
CH03	14	73	-4	11 - Right Orbitofrontal area	0.52
CH04	-18	72	10	10 - Left Frontopolar area	0.98
CH05	-25	68	15	10 - Left Frontopolar area	0.95
CH06	-8	66	28	10 - Left Frontopolar area	0.94
CH07	-16	58	38	9 - Left Dorsolateral prefrontal cortex	0.78
CH08	11	68	27	10 - Right Frontopolar area	0.97
CH09	21	72	10	10 - Right Frontopolar area	0.98
CH10	20	58	38	9 - Right Dorsolateral prefrontal cortex	0.83
CH11	28	68	16	10 - Right Frontopolar area	0.96
CH12	2	55	40	9 - Left Dorsolateral prefrontal cortex	0.91
CH13	-10	48	51	9 - Left Dorsolateral prefrontal cortex	0.84
CH14	13	48	52	9 - Right Dorsolateral prefrontal cortex	0.84
CH15	66	-19	44	1 - Right Primary Somatosensory Cortex	0.55
CH16	73	-23	7	22 - Right Superior Temporal Gyrus	0.72
CH17	70	-37	28	40 - Right Supramarginal gyrus part of Wernicke's area	0.4
CH18	57	-35	57	40 - Right Supramarginal gyrus part of Wernicke's area	0.5
CH19	61	-52	44	40 - Right Supramarginal gyrus part of Wernicke's area	0.75
CH20	49	-65	53	39 - Right Angular gyrus, part of Wernicke's area	0.68
CH21	72	-39	-10	20 - Right Inferior Temporal gyrus	0.52
CH22	68	-53	9	21 - Right Middle Temporal gyrus	0.4
CH23	62	-64	-6	37 - Right Fusiform gyrus	1
CH24	59	-66	26	39 - Right Angular gyrus, part of Wernicke's area	0.83
CH25	47	-78	36	39 - Right Angular gyrus, part of Wernicke's area	0.67
CH26	53	-79	10	19 - Right V3	0.76

Note: Some NIR observation channels may cover multiple brain regions, and this table only lists brain regions with a coincidence degree greater than 0.4.

S4 Supplementary Materials for Behavioral Outcomes

S4.1 Normality and Homogeneity of Variance Tests

The residual method was used for overall normality testing: after saving the standardized residuals from the mixed ANOVA, Shapiro-Wilk tests were conducted according to the teaching group variable. The results showed that the residuals generally followed a normal distribution (ps > .05), with only a few residuals slightly deviating from normality (p < .05). Since the absolute values of skewness and kurtosis were both less than 1, the Q-Q plot indicated that the data points were generally distributed along the diagonal line. Additionally, the Levene test for homogeneity of variances supported the homogeneity of variances across groups (ps > .05), meeting the requirements for parametric tests. Therefore, analysis of variance (ANOVA) was still employed.

S4.2 Analysis of the teaching effect of the three groups

Table. S3 ***Detailed statistical data on subjects' information***

	Instruction Strategy	*M* ± *SD*	*F*	*p*	η_P^2
	AR Teaching group	22.44 ± 2.1			
**Age**	Visual Mental Imagery Teaching group	23.15 ± 2.7	1.17	0.315	0.029
	Control group	22.15 ± 2.55			
	AR Teaching group	1.33 ± 0.48			
**Gender**	Visual Mental Imagery Teaching group	1.37 ± 0.49	0.52	0.95	0.001
	Control group	1.37 ± 0.49			
**Fondness for Pipa music**	AR Teaching group	3.52 ± 0.64	1.36	0.262	0.034
	Visual Mental Imagery Teaching group	3.26 ± 0.71			

	Control group	3.52 ± 0.64			
Level of daily exposure to Pipa music	AR Teaching group	1.96 ± 0.85	1.84	0.165	0.045
	Visual Mental Imagery Teaching group	2.41 ± 0.89			
	Control group	2.3 ± 0.91			

Note. The results showed no significant group difference.

Table. S4 ***The detailed statistic of pre-test performance***

		Instruction Strategy	$M \pm SD$	F	p	η_p^2
**Familiarity with music**	Positive music	AR Teaching group	2.46 ±1.52	0.389	0.679	0.01
		Visual Mental Imagery Teaching group	2.67 ± 1.47			
		Control group	2.81 ± 1.42			
	Negative music	AR Teaching group	2.35 ± 1.08	0.557	0.564	0.015
		Visual Mental Imagery Teaching group	2.61 ± 1.39			
		Control group	2.28 ± 1.09			
**Music Emotion Recognition Valence**	Positive music	AR Teaching group	5.26 ± 0.2	0.076	0.47	0.019
		Visual Mental Imagery Teaching group	4.94 ± 0.2			
		Control group	5.24 ± 0.2			
	Negative music	AR Teaching group	3.07 ± 0.2	1.55	0.22	0.038
		Visual Mental Imagery Teaching group	3.54 ± 0.2			
		Control group	3.15± 0.2			

**Music Emotion Recognition Arousal**	Positive music	AR Teaching group	5 ± 0.18	0.21	0.809	0.005
		Visual Mental Imagery Teaching group	4.98 ± 0.18			
		Control group	4.85 ± 0.18			
	Negative music	AR Teaching group	4.56 ± 0.17	0.466	0.629	0.012
		Visual Mental Imagery Teaching group	4.54 ± 0.17			
		Control group	4.74 ± 0.17			
**Music Emotional Experience Valence**	Positive music	AR Teaching group	5.41± 0.17	2.001	0.142	0.049
		Visual Mental Imagery Teaching group	5.07 ± 0.17			
		Control group	4.94 ± 0.17			
	Negative music	AR Teaching group	3.02 ± 0.17	2.612	0.08	0.063
		Visual Mental Imagery Teaching group	3.57 ± 0.17			
		Control group	3.3 ± 0.17			
**Musical Emotional Experience Arousal**	Positive music	AR Teaching group	5.06 ± 0.16	0.51	0.605	0.013
		Visual Mental Imagery Teaching group	5 ± 0.16			
		Control group	4.83 ± 0.16			
	Negative music	AR Teaching group	4.52 ± 0.19	0.118	0.888	0.003
		Visual Mental Imagery Teaching group	4.43 ± 0.19			

	Control group	4.56 ± 0.19

Note. The results showed no significant group difference.

S5 IBS Results Supplement

Table S5 ***Means and standard deviations of all channels of IBS during the teaching phase in the three groups（N=81）***

Variables	**AR Teaching group (*n* = 27)**		**Visual Mental Imagery Teaching group （*n* = 27）**		**Control group （*n* = 27）**	
	**Positive Music**	**Negative Music**	**Positive Music**	**Negative Music**	**Positive Music**	**Negative Music**
	M ±*SD*	*M* ±*SD*	*M* ±*SD*	*M* ±*SD*	*M* ±*SD*	*M* ±*SD*
lFPC	0.041±0.083	0.042±0.071	-0.019±0.09	-0.001±0.117	-0.032±0.078	-0.061±0.113
rFPC	0.051±0.107	0.049±0.12	-0.01±0.131	-0.014±0.128	-0.01±0.093	-0.032±0.103
lOFC	0.03±0.105	0.032±0.094	0.056±0.116	0.06±0.123	-0.058±0.128	-0.081±0.139
rOFC	0.02±0.095	0.001±0.112	0.042±0.125	0.032±0.115	-0.024±0.163	-0.064±0.18
ldlPFC	0.055±0.092	0.045±0.091	0.042±0.089	0.043±0.076	-0.016±0.09	-0.011±0.09
rdlPFC	0.057±0.11	0.044±0.111	0.034±0.1	0.031±0.104	-0.035±0.128	-0.032±0.131
rSI	0.04±0.111	0.029±0.146	0.031±0.159	0.025±0.144	-0.057±0.157	-0.023±0.174
rANG	0.072±0.08	0.05±0.091	-0.0007±0.107	0.004±0.077	-0.014±0.093	-0.002±0.071
rSMG	0.054±0.091	0.032±0.103	-0.002±0.077	-0.001±0.08	-0.027±0.092	-0.009±0.097
rITG	0.045±0.12	0.025±0.1	0.001±0.131	0.005±0.111	0.088±0.127	0.068±0.115
rTPG	0.035±0.154	0.015±0.139	-0.044±0.131	-0.018±0.13	0.0007±0.133	-0.024±0.127
rFFG	0.065±0.09	0.046±0.098	-0.01±0.117	0.003±0.119	-0.004±0.11	-0.0003±0.095
rV3	0.027±0.11	0.023±0.121	0.03±0.121	0.038±0.144	-0.019±0.14	-0.031±0.131
rSTG	-0.009±0.136	-0.024±0.152	0.028±0.14	0.005±0.147	-0.048±0.159	-0.02±0.147
